# Compression Strength Mechanisms of Low-Density Fibrous Materials

**DOI:** 10.3390/ma12030384

**Published:** 2019-01-26

**Authors:** Jukka A. Ketoja, Sara Paunonen, Petri Jetsu, Elina Pääkkönen

**Affiliations:** VTT Technical Research Centre of Finland Ltd, Solutions for Natural Resources and Environment, P. O. Box 1000, FI-02044 VTT Espoo, Finland; Sara.Paunonen@vtt.fi (S.P.); Petri.Jetsu@vtt.fi (P.J.); Elina.Paakkonen@vtt.fi (E.P.)

**Keywords:** wood fibers, regenerated fibers, nanocellulose, foam forming, compression test, stress, strain, fiber buckling, image analysis

## Abstract

In this work we challenge some earlier theoretical ideas on the strength of lightweight fiber materials by analyzing an extensive set of foam-formed fiber networks. The experimental samples included various different material densities and different types of natural and regenerated cellulose fibers. Characterization of the samples was performed by macroscopic mechanical testing, supported by simultaneous high-speed imaging of local deformations inside a fiber network. The imaging showed extremely heterogeneous deformation behavior inside a sample, with both rapidly proceeding deformation fronts and comparatively still regions. Moreover, image correlation analysis revealed frequent local fiber dislocations throughout the compression cycle, not only for low or moderate compressive strains. A new buckling theory including a statistical distribution of free-span lengths is proposed and tested against the experimental data. The theory predicts universal ratios between stresses at different compression levels for low-density random fiber networks. The mean ratio of stresses at 50% and 10% compression levels measured over 57 different trial points, 5.42 ± 0.43, agrees very well with the theoretical value of 5.374. Moreover, the model predicts well the effect of material density, and can be used in developing the properties of lightweight materials in novel applications.

## 1. Introduction

Fibrous materials are bulk media that consist of high-aspect ratio fibers of any type. Examples include paper products, textiles, insulator sheets, and fiber-reinforced composites. The current study focuses on highly porous fiber materials prepared with foam forming without added matrix polymers. Foam forming is a new technology which enables the production of ultra-light fiber networks with a density as low as 5 kg/m^3^ [1]. The strength behavior for very low densities has been analyzed in several papers [1,2,3]. However, the intermediate density range of 20 kg/m^3^ to 100 kg/m^3^, perhaps the most relevant for practical applications, has been rather poorly understood.

Compression of fibrous materials is a population phenomenon in a heterogeneous, bonded structure. A fiber with a small diameter and a high aspect ratio is its main constituent component. The entire internal structure of the fiber assembly, properties of the fibers, contacts among them, and the dimensions of the consequent network determine the response of the material under loading. As the complete analysis of all of these aspects is usually not possible, material behavior is often explained via representative idealizations. In many cases, these are based on the behavior of a single fiber under an applied load, assuming that the response of the entire fiber network is synchronized with the overall macroscopic deformation.

The uniqueness of natural wood fibers is that they bond together via several mechanisms acting on different length scales, ranging from nanoscale fibril bridges to atomistic bonds between cellulose molecules [4]. The material strength arises largely from the strength of fiber-to-fiber contacts [5]. The number of interfiber contacts and their properties (slipping, nonslipping, and breaking) are fundamental properties when considering the mechanical response of fibrous materials. The number of interfiber contacts is dominated by macroscopic material density, but it is also affected by fiber geometry and orientation. As the contact count is difficult to determine experimentally, a number of models have been proposed for varied fiber orientations. The models for planar fiber networks such as paper materials [6] do not apply to 3D networks, as the fibers are generally not aligned in-plane. Komori and Makishima [7] presented a general model for the average number of fiber–fiber contacts for arbitrary 3D fiber orientation distributions per unit volume. The model assumed a randomly built fiber mass in which two fibers overlapping each other represented a fiber–fiber contact. Later, Komori and Itoh [8] introduced steric hindrances between fibers to their model.

Analyzing the compression strength as a purely geometric (i.e., packing) problem, without any reference to local fiber deformations, is not a very fruitful approach, due to the obvious pliable nature of most fibers. Much of the earlier theoretical work has considered compression of the porous material to take place through continuous local deformations such as bending of individual fiber segments. Such an approach can be motivated by the relatively smooth macroscopic movement of the outer material surfaces. In his classic paper on wool compression, van Wyk [9] considered the bending of straight fibers that were supported at several locations along the fiber length. The average free-span length was estimated assuming a random orientation distribution of the fibers. All other phenomena, such as friction and slippage between fibers, were ignored. Much later, Maisudaira and Quin [10] limited the bending only to the first part of the compressional and recovery stress–strain curves. The second part of the compression curve was dedicated to friction between (textile) fibers and the third to mechanical properties of the (textile) fiber material. Carnaby and Pan [11] added fiber slippage as a means to accommodate the changing volume of the fiber assembly in compression, while keeping bending fibers and nonslipping contacts as the main deformation mechanism. Komori and Itoh [12,13] presented a theory to which any modes of deformation energy stored in the fiber segments may be introduced. This includes bending, axial, torsion, and shear deformation modes [14]. There are also models that discard idealized beam-bending, and consider the material only as a system of friction and spring elements [15], or rely on computer simulations of the fiber network [16,17,18,19]. Lundquist et al. [17] carried out such a simulation including the contribution of lumen collapse of hollow wood fibers at interfiber contacts. This led to a change in the relative moment of inertia facilitating the localized bending of fibers. Alimadi et al. [19], in their simulations of reconstructed and artificial networks, found strain and fiber orientation non-uniformities to contribute to strain localization and thus to overall compression response.

The goal of our study was to take a closer look at local deformations in a heterogeneous network and to explore their possible consequences in modeling of the collective material response, including the limits of simple model descriptions. The earlier theoretical models have the following problems: Firstly, assuming smooth bending as the principal deformation mode, the compression strength should behave like density to the cubic power [9,16]. In reality, the observed power is generally clearly lower, approximately 2 [20]. Secondly, a closer inspection of local deformations within a fiber network (see Section 4.1) reveals that a significant proportion of stress is released via sudden discontinuous movements of individual fibers. Earlier random fiber network models have shown that stress variation increases with decrease in the number of interfiber bonds (i.e., with decreasing density) [21]. This causes a higher mean stress in the load-bearing fiber segments for a given external load. The highly concentrated stress could trigger the experimentally observed sudden fiber movements. However, because of their discontinuous nature, following these movements in, e.g., finite element models would require extreme care so that problems with simulation stability could be avoided.

Contrary to some earlier theories for compression, we propose a significant proportion of stress release coming from the buckling of individual fibers instead of their bending. In low-density materials, collisions of free fiber segments appear to be very rare according to our visual inspection (see Section 4.1). Thus, fibers generally lack the transverse bending forces appearing in classical deformation models [9]. The load imposed by the network deformation is generally transferred to a free fiber segment at its ends. Buckling is a bifurcation phenomenon in which a local equilibrium is lost when a critical stress level is exceeded. The resulting abrupt movement of an unstable fiber can sometimes be considerable, but even a slight movement of a single fiber can redistribute local stress effectively. Buckling is often associated with instability of perfectly straight fibers, and therefore this type of mechanism could easily be omitted in natural materials containing only curved fibers. However, one should notice that the curvature of a fiber results from an uneven internal stress distribution. If the whole stability analysis were to be carried out around this curved equilibrium state including the internal stress, a similar bifurcation to the buckling of an ideal straight beam would also be defined for curved fiber segments. In fact, a similar buckling as for straight beams is also found for curved beams [22]. The critical stress levels in such beams approach those for straight beams when the so-called subtended angle decreases [22]. Thus, the simple buckling theory for straight fibers can be considered as the first approximation for fibers with low bending angles.

The work focuses on the uniaxial compression strength of a network of either natural or regenerated cellulosic fibers up to large, 50% deformations. The density range of the materials studied was relatively low, approximately from 20 kg/m^3^ to 100 kg/m^3^, which allows for large deformations to occur although the obtained compressive strength is still relatively high. As density is easy to measure, it is also a feasible variable in a developed model. The deformations seen by microscopic imaging (refer to Section 4.1) are interpreted as buckling of non-ideal (curved) fibers, which leads to changes in proposed strength formulas as developed in Section 3. The assumptions of the theory are very general, and therefore should be applicable to many kinds of random fiber networks. In this paper, the validity of the theory is tested using an extensive set of compression data for both wood fibers and regenerated viscose fibers. In Section 4, we find a perfect quantitative agreement between observed mean stress–strain behavior and the universal prediction. Because of the inherent variability of the biobased materials, such quantitative predictability of the mechanical behavior is extremely rare. Moreover, this work leads to intriguing observations regarding the role of free-span fiber segments, material density, fiber geometry, bonding, and foam properties (e.g., surfactant type) underlying the material compression behavior.

## 2. Materials and Methods

### 2.1. Materials

**Figure 1:**
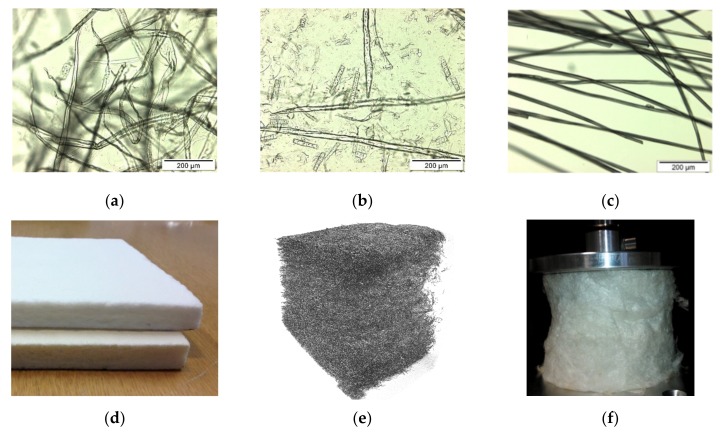
Microscopic images of the used fiber types. (**a**) bleached softwood Kraft pulp (BSKP) fibers, (**b**) chemi-thermomechanical pulp (CTMP) fibers, (**c**) viscose fibers and examples of samples produced with these fibers, (**d**) BSKP (top sheet) and CTMP (bottom sheet) samples, (**e**) tomographic image of a typical wood fiber network, and (**f**) a cylindrical viscose fiber sample with a cellulose nanofiber (CNF) binder in mechanical testing.

Several types of fiber materials were used to prepare the samples (see Figure 1). Spruce chemi-thermomechanical pulp (CTMP) with an average (length-weighted) fiber length of 1.86 ± 0.02 mm, mean width 38.2 ± 0.3 µm, and coarseness (i.e. mass per unit length) 0.20 ± 0.07 mg/m, was obtained from a Swedish pulp mill (Rottneros AB, Rottneros Mill, Sweden). CTMP fibers have typically rather circular cross-section with open lumen inside a fiber. The CTMP pulp included a significant amount (circa 30 wt %) of submicron fine particles, i.e. fines. Three variants of bleached softwood Kraft pulp (BSKP) made from spruce, obtained from a Finnish pulp mill (Metsä Fibre Oy, Äänekoski Bioproduct Mill, Finland), were also used: unrefined BSKP having a Schopper-Riegler (SR) value of 13 (code for graphs and discussion: BSKP0), refined to SR20 (BSKP1), and refined to SR36 (BSKP2). The probability for the collapse of the initially circular cross-section (i.e., closing of the lumen) increases with the refining level.

The fifth fiber material, staple viscose (Danufil, Kelheim Fibres GmbH, Kelheim, Germany), had a fiber length of 6 mm and a linear mass density of 1.7 dtex (mass in grams per 10,000 m). The fiber cross-section was approximately elliptic, with a major axis of 18.5 µm. The material was received at a moisture content of 55.3%.

Cellulose nanofibers (CNF) were used as a fine fibrous material type. These chemically unmodified nanofibers were prepared from never-dried birch Kraft pulp using a Masuko MKZA10-15J grinder (Masuko Sangyo Co., Ltd., Saitama, Japan) with three passes. The measured conductivity was 205 µS/cm at pH 7.3. The viscosity was 45 ± 3 Pa·s as obtained with a Brookfield RVDV-III+ (AMETEK Brookfield, Middleboro, MA, USA) rheometer in V73 vane spindle geometry at 10 rpm and 1.5% solids. The material was used at a solids content of 1.97%.

To foam the fiber–water suspensions, three different surfactants were used: (1) 10 wt % solution of anionic sodium dodecyl sulfate (SDS) C_12_H_25_SO_4_Na (Sigma-Aldrich, St. Louis, MO, USA) with a purity of 90%; (2) a nonionic surfactant polyethylene sorbitan monolaurate (TWEEN 20, Sigma-Aldrich, St. Louis, MO, USA); and (3) partially-saponified PVA 6-88 (approximately 88 mol % hydrolysis) hydrophilic poly(vinyl alcohol) (PVA, Kuraray, Hattersheim, Germany).

### 2.2. Sample Preparation

Two sets of samples were prepared: one with bonding wood (CTMP or BSKP) fibers and another with nonbonding viscose fibers reinforced with the CNF additive. The goal was to vary the sample density, method of fiber bonding (via wood fibers or nanocellulose), fiber stiffness and bond properties (degree of refining), and surfactant type. The differences in the forming operations between the wood fiber and viscose fiber samples affected their fiber orientations. The furnish compositions are summarized in Table 1 for bonding wood fiber samples and in Table 2 for nonbonding viscose fiber samples.

The fiber foams used to prepare the wood fiber samples (Table 1) were generated by mixing the pulp dose, foaming agent, and water in a vessel with a volume of 3–12 liters, with a mixer (Netzsch, Hedensted, Denmark) at a rotational speed of 3800 rpm to 5700 rpm. The exact dosages of water and pulp, and the foaming details depended on the target consistency (i.e., mass fraction of the solid fibers in the water suspension before mixing the foam) in the vessel, target air content, and target density. For some samples, a gradually increasing speed sequence was applied, e.g., 3800 rpm, 4200 rpm, and finally 4700 rpm. The foaming time varied between 2 min and 20 min, and the final foam volume from 3 L to 12 L. The aim was to reach an air content of approximately 60%. The wet foam was then fed along a tilted plate into a rectangular hand sheet mold, having an area of 0.0735 m^2^ (21 cm × 35 cm), and was left to drain for 20 to 40 min in ambient conditions. Thus, the structure of the original wet foam largely disappeared, and one was left with a partly collapsed random fiber network. However, the bubble size of wet foam used during forming was expected to affect the pore size distribution of this network as shown by Al-Qararah et al. [23]. Earlier studies have indicated mainly lateral fiber orientation in this type of forming procedure [24]. The sample sheets were dried overnight at 70 °C in an oven. The foaming was more effective for low pulp concentrations, which manifested as a higher sample thickness after oven drying. As an example, for the BSKP0 samples made with Tween, having a target density of 60 kg/m^3^ (and thus a grammage of 1200 gsm) and an air content of 60% of the wet foam, the thicknesses for consistencies 1%, 4%, and 6% were 4.75 cm, 3 cm, and 2.25 cm, respectively. The air content that was reached in foaming also affected thickness. In the case of air contents of 50%, 60%, and above 60% as well as the samples made with 2.84% BSKP0 pulp and SDS, and those having a target density of 40 kg/m^3^, thicknesses of 4.5 cm, 6 cm, and 7 cm after oven drying were recorded, respectively.

The dried sheets were then put on a scale and rewetted to reach a solids content of 50% by spraying the desired amount of water on the top and bottom surfaces. The moisture content was allowed to even out for 4 h (two hours lying on each side). The sample was then compressed between metal plates to the final 15 or 20 mm target thickness, guided by corner pieces having the desired height. The sample sheets were dried again overnight at 70 °C in an oven.

The viscose samples (Table 2) were prepared by first foaming water, fibers, and CNF together with Tween up to an approximate volume of 1000 mL with a mixer (Netzsch, Hedensted, Denmark) at a rotational speed of 3500 rpm. The foaming time varied between 3 and 11 min. The corresponding air content varied from 72 to 82%. The applied consistency was very high (see Table 2), and the samples V4, V5, V8, and V9 were particularly viscous foams due to the high CNF content. A rotational speed of 5500 rpm was used for them, and the required foaming time was up to 6 min. The obtained fiber foam was then poured from above into a metal cylinder (diameter 98 mm) lying on a metallic forming fabric. The resulting flow led to a more vertical fiber orientation than the orientation of the wood fiber samples [25]. Water was removed first by drainage through the bottom fabric for about 10 min, and then by drying overnight at 70 °C in an oven.

### 2.3. Sample Thickness and Weight

Thickness was recorded from the compression test at the 250 Pa load level for the wood–fiber samples and at the 106 Pa load level for the viscose fiber samples. Contrary to the wood fiber samples, the cylindrical viscose samples had a relatively uneven top. Under the load of 106 Pa, the test plate touched most of the sample top, but did not yet compress its body. The sample weight was recorded after oven drying and in standard climate before compression testing for density calculation.

### 2.4. Mechanical Testing

Cyclic compression tests of both series were run with a Lloyd LR10K universal tester (Lloyd Instruments Ltd, Bognor Regis, West Sussex, UK). The wood fiber samples (Table 1) were compressed to 10% and 50%, and the viscose fiber samples to 50% and 70% of the sample thickness. For the wood fiber series, the test speed was 10%/min for the 10% cycle and 100%/min for the subsequent 50% cycle. For the viscose series, the test speed was 100%/min for both cycles. The strain was determined based on the location of the test plate. For the current fairly thick samples this method was relatively accurate. The largest uncertainty came from an error in determining the sample thickness (i.e., origin of the strain axis) as explained above.

Five test pieces of size 5 cm × 5 cm (height 2 cm) were cut with a bandsaw (Makita Ltd, Milton Keynes, Bucks, UK) from the wood fiber sheets for testing. Each viscose sample was tested intact, but trimmed slightly after drying by cutting protruding fiber bundles with scissors along the top edge.

### 2.5. High-Speed Imaging with Correlation Analysis

The deformation mechanisms of the samples during compression were visualized using high-speed charge-coupled device (CCD) imaging (Y3, IDT, Tallahassee, FL, USA) of the side of the sample. The imaging frequency was 10 Hz with 12 µm pixel size and 1280 × 1024 pixel resolution. Because of the high imaging frequency, very strong light sources were necessary to ensure sufficient lighting to record the images. Due to the low sample density and the open porous structure, the fiber network deformations, including abrupt fiber dislocations, were detectable with this method. Surface fibers were lightly colored with a black permanent marker (Artline 100, black chisel tip). 

The responses of fibers and their shorter segments to loading were analyzed from the images both qualitatively and by using quantitative image correlation analysis [26]. In particular, we followed the cross-correlation between two subsequent frames in order to reveal rapid local structural dislocations during the compression cycle. The Image CorrelationJ plugin provided by Chinga and Syverud [26] was applied for this purpose.

## 3. Theoretical Analysis

**Figure 2:**
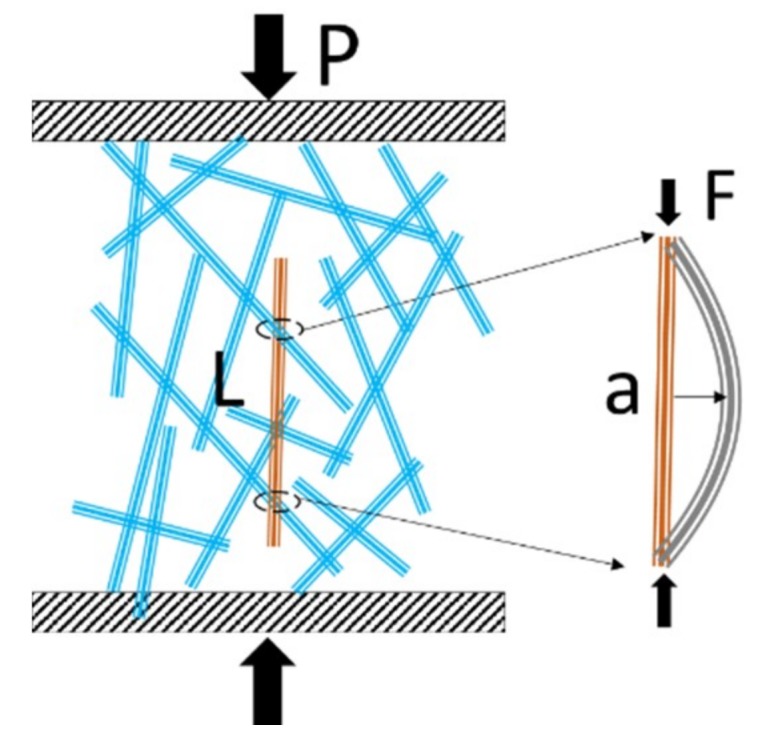
A fiber segment ranging from one joint to another in a compressed fiber network.

The possibility for local fiber buckling is not necessarily related to the orientation of the fiber with respect to the direction of macroscopic compression. Under vertical compression (see Figure 2), the fiber network also directs the stress to transverse directions due to Poisson effect, so that local buckling can take place even for fibers whose orientation differs clearly from the compression direction. Effectively, all loaded fiber segments experience a similar type of axial stress, despite their individual directions. It should also be noted that buckling is possible without failures in the interfiber bonds. Bond opening could of course be another type of sudden local deformation mechanism resulting in local stress release and finally in a gradual collapse of the network structure. We studied both mechanisms by including well and weakly bonded fiber networks in our compression experiments. 

Buckling failure of a column loaded at its ends can be described with the well-known Euler’s formula:

(1) $$F=\mu \frac{\pi^{2}EI}{a^{2}}$$

where *F* is the critical force [N], *E* is the modulus of elasticity [Pa], *I* is the minimum area moment of inertia of cross-section [m^4^], and *a* is the free-span length [m]. The factor *µ* describes the effects of boundary conditions at the ends. *µ* varies typically in the range of 1 to 4, so that the value increases with stiffer interfiber bonds. The fiber material elasticity, dimensions of the fiber cross-section, and boundary conditions are all affected by the presence or absence of a fiber lumen [17].

According to Komori and Makishima [7], the mean free-span (or segment) length can be given as

(2) $$a_{0}=\;\frac{V}{2DNLJ}$$

where *V* is the volume, *D* is the average fiber diameter, *N* is the number of fibers, *L* is the average length of fibers, and *J* is the orientation density function having the value of π/4 for isotropic fiber networks and the value of 2/π for sheet-like networks. Equation (2) can be rewritten in terms of material density ρ and fiber density $\rho_{f}$ as

(3) $$a_{0}=\frac{\pi D\rho_{f}}{8\rho J}$$

assuming a circular fiber cross-section. For the studied low-density materials, the mean free-span length $a_{0}$ became 5–40 times greater than the average fiber diameter. Moreover, we can assume that the number of interfiber contacts does not increase significantly for the applied moderate compression levels. The critical buckling load can then be expressed as a function of material and fiber properties:

(4) $$F=\mu \frac{64EIJ^{2}\rho^{2}}{D^{2}\rho_{f}^{2}}.$$

Strong square contributions to the compression strength are thus expected from material density ρ, fiber cross-section (affecting *EI*, *D*, and $\rho_{f}$), and fiber orientation (affecting J).

Even though Equation (4) describes the situation for a single fiber only, the fraction of load-carrying fibers that have not buckled for a given external stress is proportional to the mean single-fiber buckling force. Thus, the whole network strength can also be expected to depend on this load. Moreover, it is possible to develop an equation describing the stress increase as a function of the relative compression. For random fiber networks, the free-span length a is exponentially distributed [27],

(5) $$p\left(a\right)=\frac{1}{a_{0}}\exp\left(-\frac{a}{a_{0}}\right).$$

In other words, shorter spans are more frequent than longer ones, with the mean length given by Equation (3). During compression, the buckling failures are expected to appear first in the long fiber segments as the critical force is proportional to $1/a^{2}$ according to Equation (1). When the compression proceeds, the network stress increases because the remaining nonbuckled fiber segments are shorter and their total number (sharing the external load) is higher. Denoting the critical free-span length of buckling by $a_{b}$ at a certain compression level ε, the mean length of the remaining nonbuckled fiber segments becomes

(6) $$\int_{0}^{a_{b}}a\;p\left(a\right)da=a_{0}\left[1-\left(\frac{a_{b}}{a_{0}}+1\right)\exp\left(-\frac{a_{b}}{a_{0}}\right)\right]$$

The new material thickness builds up from the nonbuckled fiber segments, whereas the buckled fiber segments bend inside the open porous structure. By assuming that macroscopic dimensions scale by the same factor as the mean supporting structural element during compression, Equation (6) provides a relationship between the compressive strain ε and $a_{b}$:

(7) $$\varepsilon =\left(\frac{a_{b}}{a_{0}}+1\right)\exp\left(-\frac{a_{b}}{a_{0}}\right)$$

This would mean that the relative decay of $a_{b}$ with increasing compressive strain ε, i.e., $a_{b}/a_{0}$ = s(ϵ), would be *universal* for all low-density random fiber networks. Moreover, the relative change in compressive stress would be determined by ε quantitatively in the same way for all random fiber networks in which the buckling mechanism dominates the strength behavior. From Equations (1) and (7) we obtain, by noting that other parameters except the free-span length are equal for all strain levels,

(8) $$\sigma \left(\epsilon \right)=\frac{\sigma \left(\varepsilon_{1}\right)}{{\left[s\left(\varepsilon \right)\right]}^{2}};\;\left(s\left(\varepsilon \right)+1\right)\exp\left(-s\left(\varepsilon \right)\right)=\varepsilon$$

Essentially all other parameters except the span length are here included in the stress $\sigma \left(\varepsilon_{1}\right)$ achieved at the strain level $\varepsilon_{1}$ where the mean span length equals $a_{0}$ so that s(ε)=1.

**Figure 3:**
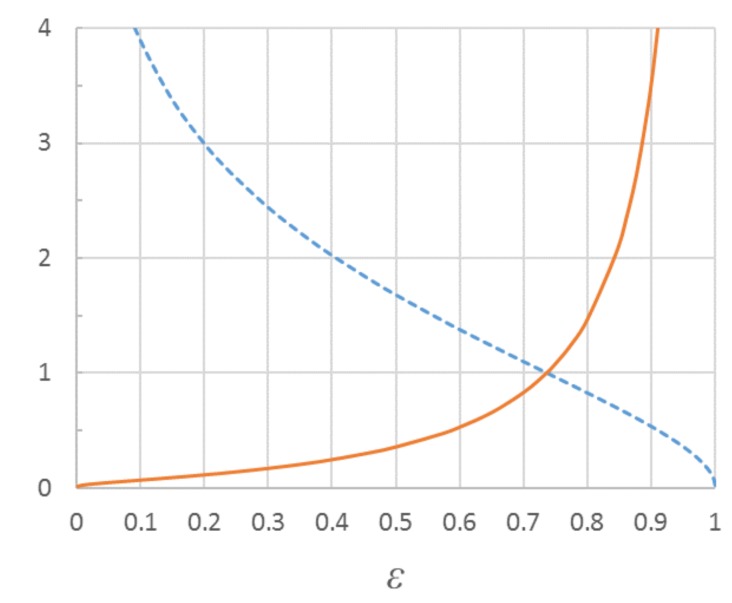
Universal functions s(ϵ) (**dashed blue curve**) and $1/{\left[s\left(\varepsilon \right)\right]}^{2}$ (**red curve**) describing the relative changes in the critical free-span length and material stress for varied compressive strain ε. The same vertical axis describes the values of both functions.

The function s(ϵ) can be implicitly solved from the above equation and is shown in Figure 3. According to Figure 3, the critical free-span length of buckling at low compression levels is much longer than the initial mean length $a_{0}$ that includes a great proportion of very short fiber segments. The critical span becomes equal to the initial mean (i.e., s(ε)=1) at a compression level as high as ε=2exp(−1)≈0.736. This value is denoted by $\epsilon_{1}$ in Equation (8). Solving Equation (8) for other values of ε provides interesting comparisons between stresses at different compression levels. For example, s(0.1)=3.890 and s(0.5)=1.678 so that the compressive stress at 50% compression is predicted to be greater than the stress at 10% compression by a factor of ${\left[s\left(0.1\right)/s\left(0.5\right)\right]}^{2}\approx 5.374$. This number turns out to be very useful for a verification of the theory in Section 4. 

There are two ways to apply the above theory which are both demonstrated in Section 4. Firstly, Equation (8) can be used to describe the stress behavior for varied compressive strains by fitting the coefficient $\sigma \left(\varepsilon_{1}\right)$ to the data so that the absolute scale of stresses is met properly. Secondly, by combining the result of Equation (8) with Equation (4), the stress is given by

(9) $$\sigma \left(\varepsilon \right)\sim \mu \frac{64EIJ^{2}\rho^{2}}{D^{2}\rho_{f}^{2}{\left[s\left(\epsilon \right)\right]}^{2}}$$

This equation can be used to describe the effect of either material density ρ or the other parameters corresponding to fiber properties. However, one should notice that the number of fibers contributing to the stress is not included in Equation (9) and should be considered separately.

The above model was studied with two sample sets made of either hollow natural wood fibers or homogeneous regenerated viscose fibers, and having primarily either a horizontal or vertical fiber orientation, respectively.

## 4. Results and Discussion

### 4.1. Observations of the Compressing Network: Stress–strain Behavior and Image Analysis

There are several possible measurement techniques to test compression properties of single fibers [28]. However, in practice it is difficult to pinpoint the initiation of a buckling failure and separate this from normal bending [28]. In the current work, the deformation of fibers and fiber segments inside highly porous compressed materials was observed with high-speed imaging and related correlation analysis. The aim was to see how heterogeneous the overall compression behavior was and to characterize some relevant deformation modes. In particular, we recorded abrupt dislocations of fiber segments inside the material network. Observed fiber bending generally led to smoother and slower deformations than these dislocations, which we interpreted as segment bucklings.

**Figure 4:**
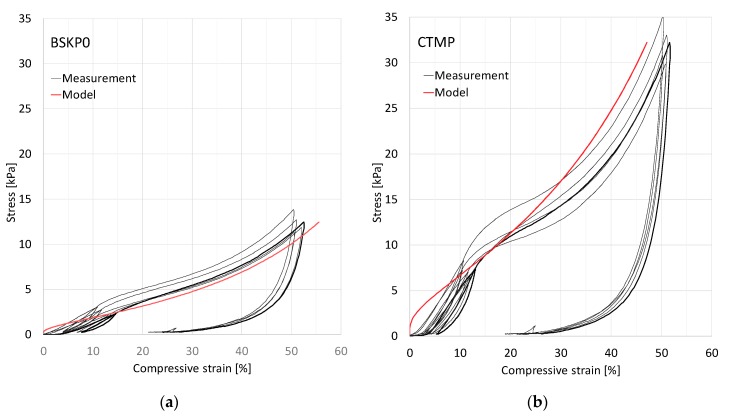
Five replicate compression tests of the trial points (**a**) BSKP0, SDS, and 37.5 kg/m^3^ and (**b**) CTMP, SDS, and 42.8 kg/m^3^. In both cases, the model (Equation (8), red curve) was fitted to the central stress value (curve shown in black) at 15% strain.

Figure 4 shows cyclic stress–strain curves for samples made of Kraft and CTMP fibers and having similar density. CTMP samples exhibited higher tensile strength and Young’s modulus due to the stiff but still well-bonding chemi-thermomechanical pulp fibers. Both curve sets show strain stiffening, which is typical for all fiber networks [14] and is described by the theoretical analysis of Section 3 based on the free-span length distribution. In particular, formation of new interfiber contacts is not required to explain strain stiffening, which contradicts a conclusion of Alimadadi et al. [19]. Moreover, the predicted stress increase with compression strain (see Equation (8)) is followed well in both cases. In fact, we compared the stress at 50% compression to that at 10% compression for a set of 57 CTMP and Kraft trial points (see Table 1) and obtained a mean ratio of 5.42 ± 0.43 (95% confidence interval) for the relative stresses over this data set. This value is well in accordance with the theoretically predicted ratio 5.374, which indicates almost perfect average agreement with our postulate on the dominance of the buckling mechanism. As the theory predicts universality along the whole compression curve, similar comparison of relative stresses could have been carried out for any other strain levels. Here we picked up the most typical ones.

**Figure 5:**
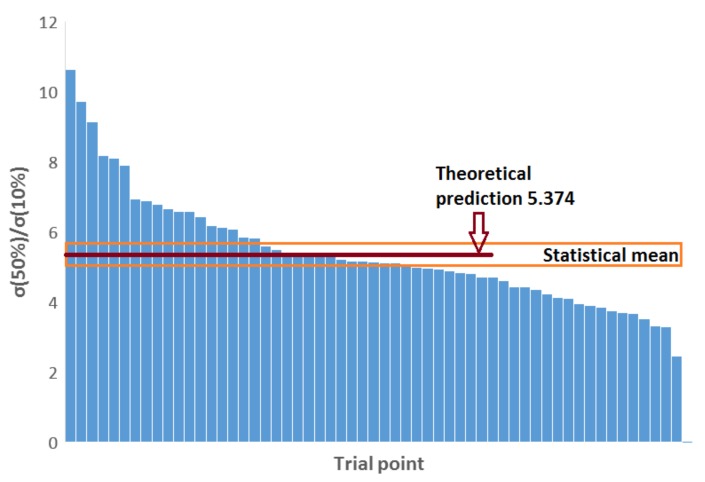
Distribution of the σ(50%)/σ(10%) ratio over the 57 trial points of Table 1 with statistical mean compared to the universal theoretical prediction.

The standard deviation of the average σ(50%)/σ(10%) ratio over the 57 trial points was 1.60 with the distribution shown in Figure 5. A significant proportion of the data points are centered in close vicinity of the universal mean value. However, there are a few cases with larger deviation from this value. There can be several reasons for the variation of the stress–compression behavior between individual trial points or samples. For example, in addition to increasing density and shortening of the free-span fiber segments, a more even distribution of the stress over the whole fiber material could also contribute to this behavior [21]. One would expect local compressions to first concentrate in the material regions that are more open than the average material structure. The foam forming process generates a much wider pore size (and thus free-span distribution) in the CTMP fiber network than in the network consisting of BSKP fibers [23]. This leads to a highly nonlinear stress–strain curve, as shown in Figure 4. The theoretical prediction of Equation (8) follows the experimental curves rather well in both cases, except at small compressive strains under 10%. For the model curves in Figure 4, the constant factor appearing in Equation (8) was not fitted at $\varepsilon_{1}$ (the measured curves did not extend that far) but at 15% compression, so that the model agreed with the central parallel measurement. The deviation at small strains could come, e.g., from fiber bending appearing at sample surfaces (also affecting the origin of the strain axis), or other factors not included in our theory.

**Figure 6:**
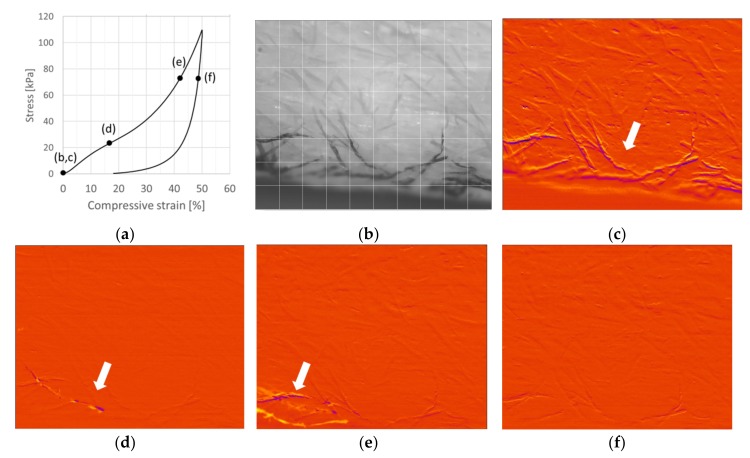
Compression test of a CTMP sample (SDS surfactant, 2% consistency, density 71.9 kg/m^3^). (**a**) Stress–strain curve for a 50% compression cycle. (**b**) Image of the network at 0% compression. Correlation plots showing fibers dislocating from previous locations (shown in yellow color) to new adjacent locations (shown in dark purple color). (**c**) Activity of the network at 0% compression (under a load of 250 Pa) Abrupt (within 100 ms) dislocations of a fiber (**d**) at 17.8% and (**e**) again in the same region at 43.9% compression. (**f**) The network during load removal at 43.9%. The grid size is 0.67 mm × 0.67 mm with similar in-depth focus length. Imaging frequency is 10 Hz.

The behavior of a CTMP network along a 50% compression cycle is presented in Figure 6. The duration of the compression part is 27.3 s, and it is represented with 264 image frames. As the image acquisition was manually triggered, it was slightly delayed from the actual start of the compression. The sample was standing on a plate, and the camera was directed to its outer surface directly above the plate. Because of the very open porous structure, most of the imaged fibers were located inside the material rather than at the immediate outer surface. As an overview, the deformation appeared to be affine [14] and smooth, without major events. During compression of the fiber network, distinctive events of any type appeared at first to be rather rare by simple visual inspection. However, the volume of the region that was followed was rather small. The x−y window size was 6.7 mm × 5.4 mm, whereas in the depth direction, the focus length was clearly below 1 mm. For affine deformation, the vertical movement of a fiber segment in the upper part of this window within one frame was expected to be 0.5 × 5.4/264 mm = 10 µm, i.e., less than the fiber radius. Occasionally, a section of an individual fiber was abruptly dislocated much more within one frame (i.e., during 100 ms). Within the studied small region, eleven of such events were easily pinpointed at compression levels of 14.4%, 17.8%, 31.4%, 37.5%, 38.3%, 38.8%, 39.0%, 40.0%, 41.1%, 43.9%, and 44.1%. Typically one to four fibers participated in these events. Only at the very end of the cycle, e.g., at 39.4% and 47.3%, could significant bending or bulging of individual fibers be observed. 

The obtained images were analyzed further using the image correlation technique. By calculation of local cross-correlation at each position in two subsequent images, movements of fibers can be detected in the whole image area. In this way, many more fiber dislocations could be observed than those events listed above, see Figure 6. In addition to the above large dislocations, the image correlation technique revealed similar smaller events in most of the studied frames. Typically, one or a few fibers had significant abrupt movement between subsequent frames (differentiated as a color change), whereas the behavior was smooth for the rest of the fiber network. The smooth behavior was indicated by the orange color corresponding to well correlating subsequent frames at the corresponding location of the network. Similar dislocations were found in all fiber orientations. There were a few frames in which practically the whole studied region was still (shown as an orange region in the correlation plots), and in some frames a number of fibers appeared to be moving suddenly. However, it was easiest to observe dislocations near the bottom region, where the colored fibers were located. Interestingly, practically no similar dislocations were observed when letting the network relax by moving out the external load after the peak point of Figure 6a.

**Figure 7:**
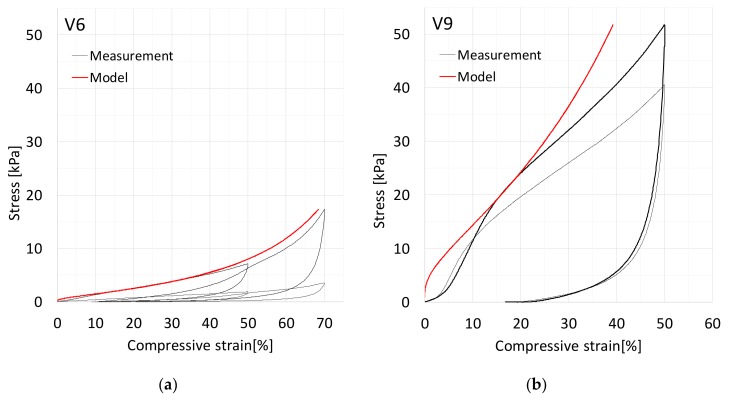
Cyclic compression test of (**a**) three replicates of V6 and (**b**) two replicates of V9 (only 50% cycle). The model (Equation (8), red curve) was fitted at 15% compressive strain to the test shown in black.

Figure 7 shows cyclic stress–strain curves for viscose samples V6 and V9. The stress–compression behavior is again followed well, especially at the lower CNF-content, for which the basic assumptions of the theory on the role of the free-span lengths are likely to be valid. As shown in Figure 7a, the 2nd cycle produces a lower stress than the 1st cycle due to the buckled segments until the maximal strain of the 1st cycle is achieved. Beyond this 50% strain level, new segment bucklings are required so that the stress rises and continues the trend set by the 1st cycle. The formed envelope of the two cycles is nicely predicted by the theory of Section 3. The higher CNF-content in Sample V9 leads to greater stress already at intermediate strain levels in Figure 7b. This suggests that the interfiber contacts created by nanocellulose are rather stiff and do not break appreciably in this sample. Moreover, it is probable that nanocellulose forms small membranes with their own substructure inside the fiber network at the high CNF content [29].

**Figure 8:**
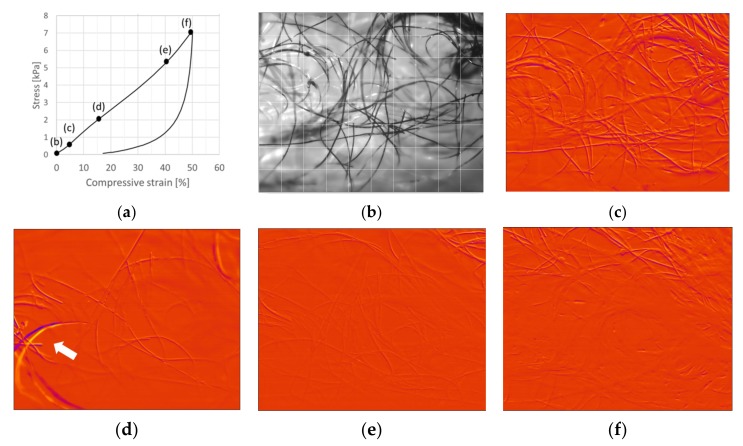
Compression test of the viscose sample V6. (**a**) Stress–strain curve for a 50% compression cycle. (**b**) Image of the network at 0% compression. Correlation plots showing dislocations of fibers from previous locations (shown in yellow color) to new adjacent locations (shown in dark purple color). (**c**) Dislocation activity in the network at low 5.6% compression. (**d**) Abrupt strong fiber dislocation at 17.9% compression; (**e**) The almost-still network at 41.8% compression. An almost identical correlation plot was also observed at 41.8% in release. (**f**) Dislocations concentrating on the upper region of the image at 49.6% compression. Imaging frequency: 10 Hz.

**Figure 9:**
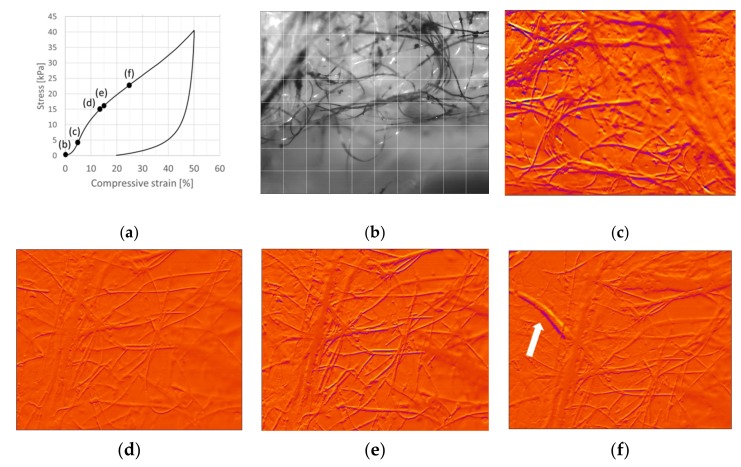
Compression test of the viscose sample V9. (**a**) Stress–strain curve for a 50% compression cycle. (**b**) Image of the network at 0% compression. Correlation plots showing dislocations of fibers from previous locations (shown in yellow color) to new adjacent locations (shown in dark purple color). (**c**) Intense and large dislocations in the network at 4.6%. (**d**) The network showing moderate and evenly distributed dislocations at 13.5% compression. (**e**) After 0.8 s at 14.5% compression, the dislocations intensified again. (**f**) An abrupt large fiber dislocation at 25.1% compression. Imaging frequency: 10 Hz.

Figure 8 and Figure 9 show the 50% compression cycle for viscose sample V6, with a CNF-content of 1.0, and for V9, with a CNF-content of 5.0, together with snapshots of the network from the compression part. The high amount of CNF can be seen in the images of V9 as dark spots.

Deformations of low-density random fiber networks have been shown to be non-affine [14]. This means that local deformation fields fluctuate around an average expansion or compression behavior. With decreasing density, non-affine deformation tendency becomes more noticeable. During testing of the thick (or high) viscose fiber samples, we also observed rather large deformation fronts on the sample surface. Similar uneven progression of the compression was observed in the videos. For example, the region in the upper right corner of V6 was compressing faster at the average 30% to 50% compression levels than the other areas in the image.

Abrupt and countable dislocations of fibers occurring in 100 ms, i.e., between two consecutive frames were first visually counted from the recorded image sequences for samples V6 and V9. For V6 (see Figure 8), dislocations of fibers, fiber groups, or fiber sections were easily seen at 4.3%, 7.5%, 17.5%, 18.0%, 19.3%, 30.4%, 31.3%, 37.3%, 38.2%, 38.4%, and 40.0% displacements. The correlation plots show many more dislocations of fibers. The dislocation activity in the network is high at low 5.6% compression, see Figure 8c. This agrees with the earlier claims on the dominance of buckling failures at low compression levels. However, bucklings are frequent throughout the increasing compression cycle. For example, an abrupt strong fiber dislocation was observed at 17.9% compression (Figure 8d), together with several smaller fiber movements at the same time. On the other hand, all fibers stayed still or moved smoothly occasionally, see Figure 8e (41.8% compression). For the sample V6, the dislocations were rather evenly distributed over the whole studied region. Figure 8f shows an example in which the dislocations concentrate on an upper part at 49.6% compression.

With increasing CNF content, the network becomes more connected and the response more collective, as shown in Figure 9 for sample V9. However, it should be noted that often some fibers move much more than others for certain frames (e.g., see Figure 9c,f). Despite the high dislocation activity throughout the increasing compression cycle, rather high maximum stress was still achieved (see Figure 7b). The high connectivity means that a large population of all fibers contributes to load bearing, which improves the material strength.

### 4.2. Regression Model for Bonding Fibers

Mechanical CTMP pulp contains fibers with a wide length distribution as well as fines. The surface of the wood fibers is fibrillated due to the mechanical forces of the pulping process. In addition, CTMP fibers have different cellulose crystal structure and bending stiffness compared to regenerated cellulose fibers [25]. Both fiber types are hydrophilic, but their bonding properties differ considerably. Whereas the CTMP fibers and wood fines bond strongly via hydrogen bonding and other short-range forces, viscose fibers have low bonding ability. On the other hand, strong-bonding Kraft fibers are more flexible than CTMP fibers and affect the mechanical properties of the Kraft fiber networks.

The speed and duration of mixing and the various geometries of the mixer setup, such as propeller shape, all have an effect on the obtained foam. For the wood fiber series, only the mixing time varied considerably. The target air content was between 50% and 60%. Especially for samples foamed with PVA, this level was not reached, and the air content remained at a lower level ranging from 40% to 50%.

The sample preparation was aimed at given densities (40 kg/m^3^, 60 kg/m^3^, and 80 kg/m^3^) and constant grammages (600 gsm, 800 gsm, 1200 gsm, and 1600 gsm). The 40 kg/m^3^ sample set contained the refined Kraft pulp raw materials. The sample thickness after oven drying (i.e., before compression) was clearly affected by surfactant type, and by pulp consistency in the mixing vessel. The smaller the consistency was, the easier it was to foam up the stock volume (leading to higher air content), and the higher was the resulting sample thickness after oven drying. The greatest drop in thickness was experienced between consistencies 1% and 2% for the unrefined BSKP0 samples. Almost invariably, SDS gave the highest thicknesses, followed by Tween and PVA. The distances that the samples had to be compressed to reach the 20 mm target thickness ranged from 70 mm, especially for samples prepared with SDS, to only 2.5 mm. The average final density and standard deviation for the samples (39 pcs) that targeted at 40 kg/m^3^ was 44.2 ± 4.2 kg/m^3^, for the 60 kg/m^3^ samples (12 pcs) 66.2 ± 5.6 kg/m^3^, and for the 80 kg/m^3^ samples (6 pcs) 84.1 ± 11.0 kg/m^3^.

**Figure 10:**
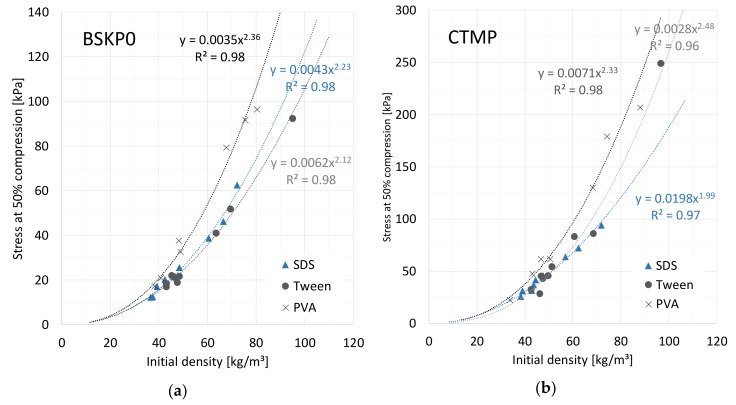
Maximum stress at 50% compression as a function of initial density for (**a**) all BSKP0 and (**b**) all CTMP samples made with three different surfactants. The curves are power-law fittings to the data.

**Figure 11:**
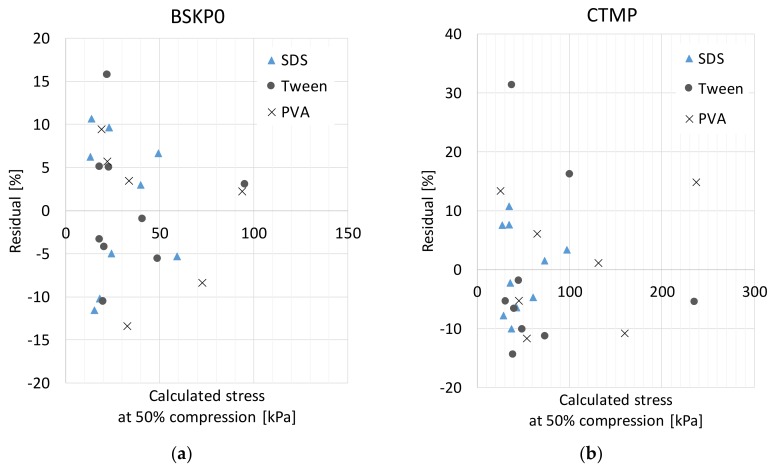
Residuals of fitted regression models at 50% compression for (**a**) BSKP0 and (**b**) CTMP for three different surfactants.

Figure 10 shows the stresses at the 50% compression level. Equation (9) predicts a square dependence of the stress on the initial density at any strain level. Motivated by this fact, we plotted stress against the initial density and fitted power curves to the results for each surfactant separately (Figure 10). All the data points appeared to follow their corresponding curves relatively closely. The density dependence was described with a power varying between 1.99 and 2.48 for the studied cases. The fact that the powers were rather close to 2 further supports the assumption that buckling is a dominant deformation mode underlying network strength, rather than fiber bending (corresponding to cubic dependence on density). The slight deviations from the square behavior could result from collective phenomena, such as distribution of bond properties that are not described by the idealized average model. The regression model predictions compared to the measured values are shown in Figure 11. The residuals were relatively random, confirming the good fit of the regression models. However, the predictions appeared to be somewhat poorer at low stress values.

The stress levels were generally higher for CTMP compared to BSKP0. These fibers differ mainly in coarseness and openness of lumen. The difference in bending stiffness for the CTMP and Kraft fibers is described by the factor *EI* in Equation (9). This factor is expected to be significantly higher for CTMP than for Kraft [30]. On the other hand, a given density corresponds to a higher number of Kraft than CTMP fibers because of the lower coarseness (typically 0.14−0.18 mg/m [31,32]) of Kraft. It is difficult to sum up these factors into a reliable strength estimate, as precise values of the various parameters are not known. However, with the factor 1.5 difference in coarseness, together with a factor-of-three difference in dry bending stiffness (for wet fibers this difference would be larger [30] because of higher moisture content in Kraft), Equation (9) predicts a similar difference in compression strength to that seen in Figure 10.

General fibrous materials exhibit rigidity percolation, a critical density at which the structure acquires stiffness [3,14]. The threshold density is affected by, e.g., the nature of the fiber-to-fiber contacts, fiber orientation, and fiber bending stiffness. Below this density, Young’s and shear moduli are both considered to be zero. For low-density 3D wood–fiber networks, Burke et al. [3] recently found the critical density to vary linearly with respect to the initial liquid fraction of foam used to form the structure. The obtained values varied in the range of 3 to 8 kg/m^3^. Looking at their data (Figures 7 and 8 of Burke et al. [3]), our theoretical model (Equation (8)) appears also to describe their stress–strain behavior reasonably well at 15 kg/m^3^. The same holds at high strains for the case studied by Alimadadi and Uesaka ([1], Figure 11a; see also Figure 15 of Alimadadi et al. [19]) at as low a material density as 5 kg/m^3^. On the other hand, the current theory does not explain the linear dependence of the compressive modulus on density observed by Burke et al. [3], because the modulus was determined at very low strains beyond the validity of our model.

**Figure 12:**
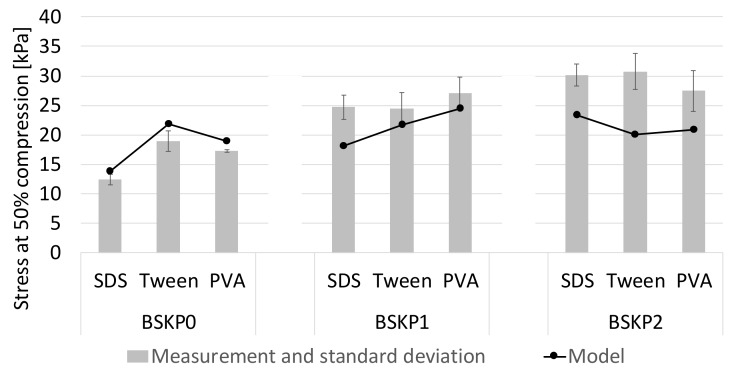
Estimation of the maximum stress at 50% compression for the BSKP1 and BSKP2 samples based on the regression model for BSKP0.

The regression models for unrefined BSKP0 samples were also applied to the refined samples (Figure 12). Refining imparts damage, such as kinks, compressions, and delamination to fiber walls, and thus improves the bonding capacity of wood fibers. The effect can be seen as increasing compression stress, irrespective of the surfactant. The model fitted to the BSKP0 data could not capture this effect, and the estimation started to lag behind the measurements for BSKP1 and BSKP2. According to Rusu et al. [33], the main contributing factor to fiber (FiberMaster) bendability is internal fibrillation rather than cross-sectional geometry (or moment of inertia). Internal fibrillation reduces the bending stiffness of a fiber wall, causes fiber collapse, and thus changes the boundary conditions in Equation (9).

### 4.3. Regression Model for Nonbonding Fibers

Regenerated viscose fibers from cellulose have considerably different structure and properties compared to natural wood fibers. Viscose fibers are manufactured as a single straight filament and are cut to length, generally leading to a smooth surface and uniform internal structure and cross-section. The viscose fibers show practically no bonding due to the very small contact area between their rounded surfaces. External bonding material, such as nanocellulose, is needed for the fiber assembly to maintain its integrity. By varying the absolute and relative amounts of binding material, the effects of fiber contact number and strength can be studied.

**Figure 13:**
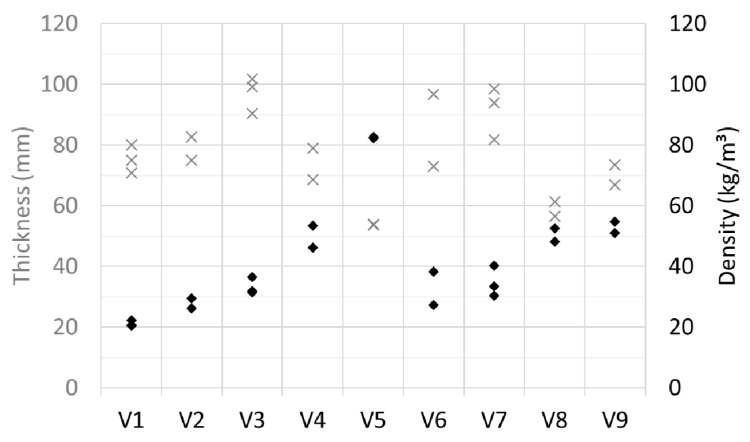
Thickness (crosses) and density (diamonds) of the viscose samples.

The preparation of the nonbonding viscose material did not aim at a predetermined density. Therefore, the sample densities ranged from 20 kg/m^3^ to 80 kg/m^3^ (see Figure 13). In certain conditions, such as a certain raw material consistency range, the air content of the batch can be independently chosen as an endpoint for the mixing (foaming). In higher consistencies, foaming does not occur unless additional energy is introduced to the stock, or mixing duration is increased. For viscose samples, the consistencies were an order of magnitude higher compared to the wood fiber samples. Samples V4, V5, V8, and V9 were relatively hard and not easily pliable to compression. The lightest samples were very open arrays of fibers, and the least bound samples, particularly samples V6 and V7, easily disintegrated during handling.

**Figure 14:**
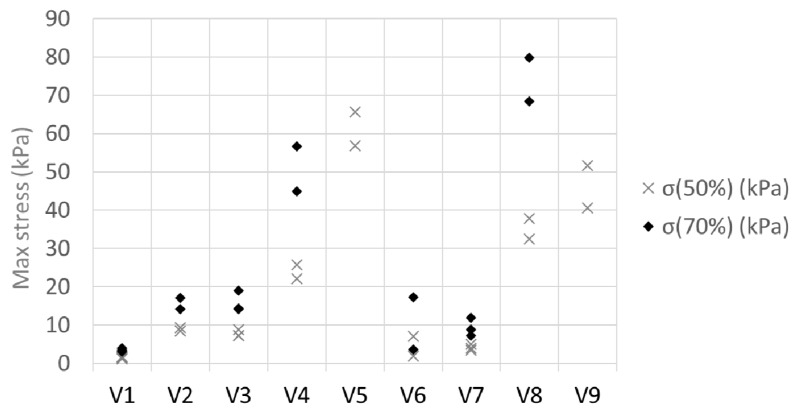
Maximum stress during 50% and 70% compression cycles.

Figure 14 summarizes the compression test results. The stresses increased as a function of density for both the constant and the varying CNF ratio series. It was not possible to compress samples V5 and V9 to 70% with the 1 kN load cell that was used.

**Figure 15:**
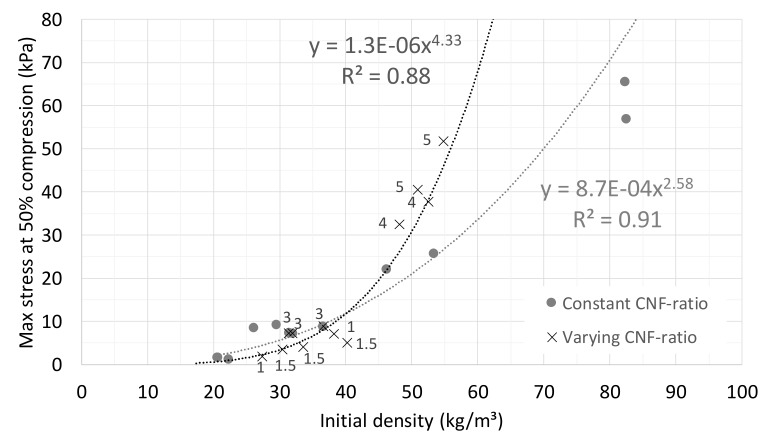
Maximum stress at 50% compression as a function of initial density for viscose samples. CNF ratios given as data point labels [%]. The curves are power-law fittings to the data.

The stresses at 50% compression were then plotted against the initial density, and a regression model was fitted to the data (see Figure 15). It is evident that the behavior is different for the two series. The samples with constant CNF ratio had an increasing number of fiber contacts that were reinforced with equal shares of CNF fibers. In the regression model, the power exponent 2.58 was comparable to that for the wood fiber series, indicating a similar deformation mechanism to that described earlier in Section 3. However, for the series with an increasing CNF ratio, both the number of fiber contacts and the relative amount of CNF reinforcing the contacts were increasing. The exponent 4.33 was considerably higher than for wood fiber samples. The difference reflects the potential of nanocellulose to form strong substructures in addition to strengthening the interfiber bonds. With increasing CNF concentration, a greater proportion of CNF forms membranes with a thickness of typically a couple of micrometers [29]. These give additional strength to the material, which is not described by our buckling model.

**Figure 16:**
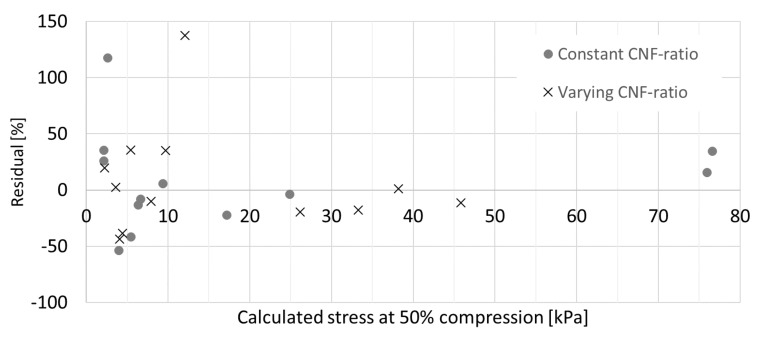
Residuals of fitted regression models at 50% compression for the viscose samples at constant and varying CNF ratios.

The quality of the prediction obtained with regression curves is described by a residual plot shown in Figure 16. Especially at lower stresses, the residuals are much higher than for the bonding fiber cases.

## 5. Conclusions

In this paper we developed a new theoretical model for the compression strength of low-density random fiber networks assuming that buckling of fiber segments dominates the strength behavior throughout the compression curve. The general theory was tested against a vast set of foam-formed fiber materials in the density range of 20 kg/m^3^ to 100 kg/m^3^. This comparison led to the following conclusions.

- The mean compression–stress behavior is described by a quantitatively universal curve, along which the relative changes do not depend on the fiber properties (assuming well-bonded fibers). Such quantitative predictability is not generally expected for biobased materials with inherent variability. In this case, the (statistical) universality comes from the dominance of exponentially distributed free-span lengths at high compression levels.
- The effect of material density is described by correlation curves with a power that is close to the theoretical prediction of 2 in the case of natural fibers. For nonbonding fibers, the same holds at low amounts of the CNF binder. With increasing CNF amount, deviations from the theory are seen, probably because of additional membrane substructures formed within the fiber network.
- Image analysis shows abrupt fiber dislocations throughout the compression cycle and thus confirms the basic assumption of our theory.

In principle, our buckling theory is generally valid for well-bonded random fiber networks with low density. Thus, in addition to biobased materials, the theory should be applicable, e.g., to porous fiber metals [34] and aerogels [35], for which very similar compression–stress behavior can be observed as for the foam-formed fiber materials studied here. We expect similar statistical universality as described in this work to exist for many other types of random fiber networks. The universality class is defined by the statistical distribution of free segment lengths. In order to observe the quantitative universality, data needs to be collected over a large amount of experimental samples as there may be variations among individual samples due to surfaces, local density variations, and other features not included in the theory. In this work, the theoretical prediction appeared applicable throughout the studied density range. It will be interesting to explore the validity of the theory at higher material densities as well. 

## Figures and Tables

**Table 1:** Composition of wood pulp samples. Dosages of surfactants: PVA 3 g/L; SDS 1 g/L; and Tween 6.5 g/L. Total number of trial points is 57. One 21 cm × 35 cm plate-like sample was prepared for each of them from which five parallel test pieces were cut.

Fiber Material	Suspension	PVA	SDS	Tween
Pulp Type	Consistency [%]	Density [kg/m^3^]	Density [kg/m^3^]	Density [kg/m^3^]
BSKP0	1.00	40.9	47.3	43.2
	2.00	48.3	42.6	43.2
	2.84	38.3, 80.4	36.6, 37.5, 39.2, 72.2	45.2, 46, 47.5, 95.1
	3.00	49, 67.8	48.5, 66.5	48.4, 63.6
	4.00	75.5	60.5	69.5
BSKP1	2.13	42.7	42.4	47.3
BSKP2	2.36	39.9	47.6	45.5
CTMP	1.00	46.7	42.9	46.8
	2.00	33.7, 43.2, 88.1	39, 42.8, 47.6, 43.6, 71.9	42.6, 47.5, 49.6, 51.3, 96.7
	3.00	50.6, 68.3	44.5, 62.3	46.2, 60.6
	4.00	74.3	38.3, 56.9	68.5

**Table 2:** Composition of the viscose samples. Dosage of surfactant (Tween 10%) 18 mL. Amount of water 0.17 L.

ID	Viscose [g]	CNF [mL]	CNF Content [dry-wt %]	Consistency [%]	Initial Air Content [%]	Density [kg/m^3^]
V1 ^1^	23	18	3.0	11.9	81	21.1
V2	34.5	27	3.0	16.9	74	27.8
V3 ^1^	46	36	3.0	21.3	77	33.3
V4	57.5	45	3.0	25.3	66	49.8
V5	69	54	3.0	28.9	47	82.4
V6	46	12	1.0	21.3	78	32.8
V7 ^1^	46	18	1.5	21.3	79	34.8
V3 ^1^	46	36	3.0	21.3	77	33.3
V8	46	48	4.0	21.3	71	50.3
V9	46	60	5.0	21.3	68	52.9

^1^ Three replicates. Otherwise two replicates. Series with constant CNF content: V1 to V5. Varying CNF content: V6, V7, V3, V8, and V9. V3 belongs to both series.
